# A Challenging Case of Wilson's Disease

**DOI:** 10.7759/cureus.42655

**Published:** 2023-07-29

**Authors:** Rita João Soares, Nuno Monteiro, João Machado, Joana Silva Marques, Ana Nunes

**Affiliations:** 1 Department of Internal Medicine, Centro Hospitalar Tondela-Viseu, Viseu, PRT

**Keywords:** chelation, atp7b, ceruloplasmin, liver, copper, wilson's disease

## Abstract

Wilson's disease (WD) is an inherited disorder characterized by the accumulation of copper in various organs, particularly the liver, central nervous system, and cornea. The clinical presentation of WD can vary widely. Diagnosis requires a combination of clinical and biochemical findings. We present a case of a 20-year-old woman who presented to the Emergency Room with progressive motor decline. She exhibited characteristic neurological symptoms and signs, such as hypomimia, bradyphrenia, bradykinesia, dysarthria, sialorrhea, upper limb dystonia, and wing-beating tremor. Ophthalmological examination revealed corneal deposits known as Kayser-Fleischer rings. Laboratory investigations demonstrated low levels of ceruloplasmin and elevated serum copper. Brain MRI showed typical signs of copper deposition in the basal ganglia. The Leipzig criteria were used to confirm the diagnosis. Treatment with penicillamine and zinc acetate resulted in symptom improvement. This case highlights the diverse presentation of WD and the importance of early diagnosis and prompt treatment initiation.

## Introduction

Wilson's disease (WD) is an inherited disorder caused by a defect in the ATP7B gene, resulting in impaired copper transport in hepatocytes and subsequent copper accumulation in various organs, particularly the liver, central nervous system, and cornea [1-4]. The clinical presentation of WD is diverse. Psychiatric manifestations often serve as the initial symptom, but they frequently do not lead to a diagnosis until neurologic or hepatic symptoms emerge. Symptoms related to hepatic dysfunction in WD can vary from an asymptomatic increase in liver enzyme levels to fulminant liver failure. Typically, during the early stages of the disease, there is a mild elevation in transaminases, which progresses to chronic active hepatitis, followed by fibrosis and eventually cirrhosis. The most common symptoms observed at the time of presentation include jaundice, anorexia, and emesis [3,5]. Diagnosis of WD relies on a combination of clinical features and biochemical markers. Evaluation of hepatic function, including assessment of disease severity and presence of cirrhosis, is crucial. Imaging modalities like computed tomography (CT) or magnetic resonance imaging (MRI) can reveal typical signs of cirrhosis [1]. Brain MRI may show bilateral long T1 and T2 signals in the basal ganglia, indicative of copper deposition [6]. The Leipzig criteria provide a method for diagnosing WD based on clinical and laboratory data. The criteria involve a combination of clinical examination and laboratory findings, and a diagnosis is confirmed if a minimum of four points is obtained. Although primarily designed for research purposes, these criteria can also be helpful for physicians in clinical practice, especially when genetic testing is not readily available [3].

## Case presentation

**Figure 1:**
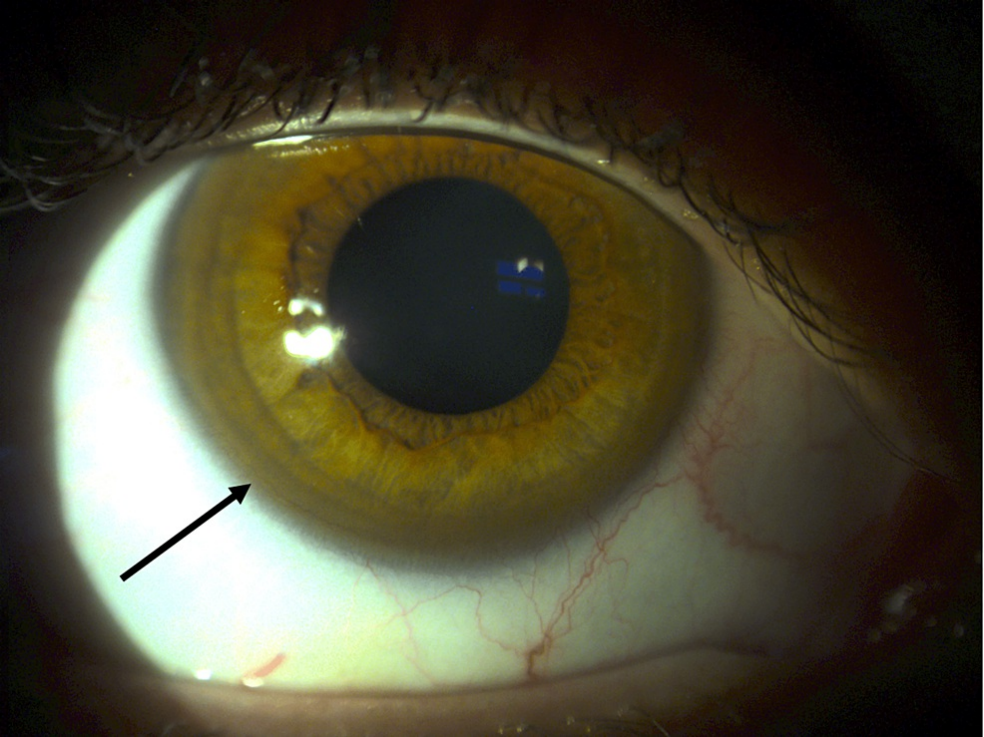
Kayser-Fleischer (KF) ring (arrow). Evaluation of KF ring was done by naked eye examination using torch light.

**Figure 2:**
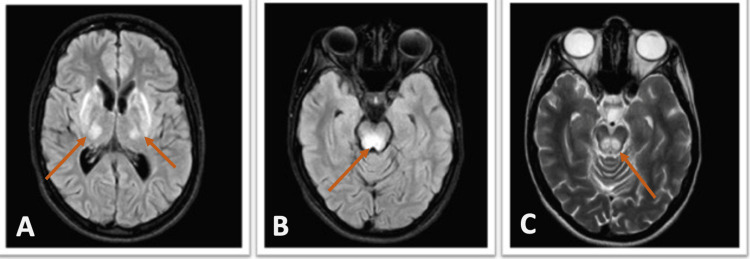
Patient's brain MRI. (A) T2 FLAIR images revealed symmetrical hypersignal in the thalami, lentiform nuclei, and lateral aspects of the brainstem. (B, C) Axial T2 FLAIR images showed hypersignal in the tegmental and mesencephalic roof.

**Figure 3:**
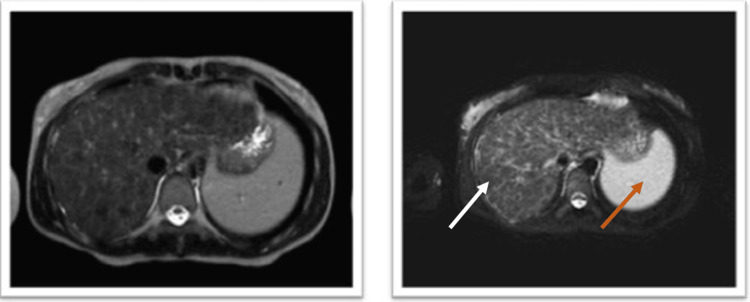
Patient's abdominal MRI revealing a liver with diffuse heterogenic nodular structure (white arrow) and mild splenomegaly (orange arrow).

A 20-year-old woman presented to the Emergency Room with progressive motor decline over several months. She reported uncontrolled upper limb tremors, anorexia, and episodes of lipothymia. Initially, her symptoms were attributed to her psychiatric condition related to a history of sexual abuse. Despite psychiatric treatment, her symptoms continued to worsen. On examination, the patient exhibited hypomimia, bradyphrenia, bradykinesia, severe dysarthria, sialorrhea, dystonia of the upper limbs (more pronounced in the left arm), muscle stiffness, and wing-beating tremor. The ophthalmological evaluation revealed corneal deposits consistent with Kayser-Fleischer rings (Figure 1). Based on clinical suspicion, the patient was admitted with a probable diagnosis of Wilson's disease (WD). Subsequently, she underwent additional investigations, and laboratory tests revealed low levels of ceruloplasmin. (3.62 mg/dL; reference range: 22-58 mg/dL) and elevated serum copper (29 μg/dL). The urinary copper excretion rate was within the normal range (32 μg/24h). Brain MRI demonstrated extensive hypersignals in the caudate nuclei, putamina, thalamus, cerebral peduncle, mesencephalon, and protuberance, along with characteristic hypointensities in the capsular striate regions (Figure 2). Abdominal MRI revealed a liver with diffuse heterogenic nodular structure and mild splenomegaly (Figure 3). A diagnosis of WD was established using Leipzig's Eighth International Meeting on Wilson's disease score, which accounted for severe neurological symptoms and brain MRI findings (2 points), the presence of Kayser-Fleischer rings (2 points), low ceruloplasmin level (2 points) and elevated serum copper (1 point), making a total score of 7 points [1,3]. Treatment was initiated with increasing doses of penicillamine and zinc acetate. The patient showed symptom improvement and a daily dose of 900 mg of penicillamine was achieved without hepatic function deterioration. A low copper diet was prescribed, and the patient underwent physical rehabilitation, resulting in significant symptomatic improvement. At discharge, her dystonia and tremors were reduced, and she could walk without assistance. Follow-up showed continued improvement, with the patient regaining the ability to run and feed herself. Urinary copper excretion increased to values well above the normal threshold (938 μg/24h and 1894 μg/24h at one and two months, respectively) under penicillamine treatment. Treatment with penicillamine at the maximum dose was maintained, with regular monitoring of hepatic function.

## Discussion

In WD, approximately 40% of patients initially present with hepatic manifestations, while 40%-50% present with neurological symptoms, and 10% primarily exhibit psychiatric symptoms. Hepatic manifestations can range from asymptomatic to acute hepatic failure [5]. Half of the patients with hepatic presentations have Kayser-Fleischer rings. In this case, the patient presented only with psychiatric and neurological symptoms. It is challenging to determine whether the psychiatric symptoms preceded the neurological symptoms or if they were solely attributed to the disease, given the patient's history of trauma. However, the presence of typical neurological signs and Keyser-Fleischer rings during the Emergency Room evaluation raised suspicion of WD. Typical laboratory findings in WD include increased hepatic copper concentration, increased non-ceruloplasmin-bound copper concentration, and low plasma ceruloplasmin levels. In this case, the patient had a low ceruloplasmin level and elevated serum copper, resulting in an estimated non-ceruloplasmin-bound copper concentration of 17.6 μg/dL, which alone was insufficient for a definitive diagnosis. The Leipzig score was utilized to assist in the diagnosis of WD, considering the neurological symptoms, brain MRI findings, presence of Kayser-Fleischer rings, low ceruloplasmin level, and elevated serum copper, confirming the diagnosis [1]. Although a urinary copper excretion rate of >100 μg/24h is commonly observed in WD [7], the patient's rate was within the normal range. However, urinary copper excretion was used to monitor treatment response, with values exceeding the normal threshold at one and two months. This, along with objective neurological improvement, supported the continuation of penicillamine treatment, the only available chelator in the patient's center. WD continues to be a vibrant and dynamic field of research. In recent years, significant progress has been made in exploring novel therapeutic approaches, including the investigation of new copper chelators such as methanobactins and DMP-1001. Additionally, orthotopic liver transplantation and gene repair have emerged as potential treatment modalities for this condition [8]. However, continued research efforts are necessary to advance our knowledge and improve outcomes for individuals affected by this disease.

## Conclusions

WD is a complex and rare autosomal recessive disorder that disrupts copper metabolism and affects individuals worldwide. Early detection and diagnosis are crucial in managing the disease effectively, as timely intervention can prevent irreversible complications to vital organs such as the liver and brain. The purpose of this case report is to raise awareness about the importance of considering WD as a potential diagnosis in patients with a history of neurologic symptoms.

## References

[1] Członkowska A, Litwin T, Dusek P (2018). Wilson disease. Nat Rev Dis Primers.

[2] Ferenci P, Caca K, Loudianos G (2003). Diagnosis and phenotypic classification of Wilson disease. Liver Int.

[3] Mulligan C, Bronstein JM (2020). Wilson disease: an overview and approach to management. Neurol Clin.

[4] Guindi M (2019). Wilson disease. Semin Diagn Pathol.

[5] Hedera P (2019). Wilson's disease: A master of disguise. Parkinsonism Relat Disord.

[6] Yu XE, Gao S, Yang RM, Han YZ (2019). MR imaging of the brain in neurologic Wilson disease. AJNR Am J Neuroradiol.

[7] Pfeiffenberger J, Lohse CM, Gotthardt D (2019). Long-term evaluation of urinary copper excretion and non-caeruloplasmin associated copper in Wilson disease patients under medical treatment. J Inherit Metab Dis.

[8] Aggarwal A, Bhatt M (2020). Wilson disease. Curr Opin Neurol.

